# Draft genome sequence of a *Bacillus cereus* isolated from a tilapia tank in Alberta, Canada

**DOI:** 10.1128/mra.00968-25

**Published:** 2026-02-27

**Authors:** Xiaoji Liu, Scott Hrycauk, Mruthula Narayan, Skyler Umlah

**Affiliations:** 1 Agriculture and Agri-Food Canada, Lacombe Research and Development Centre 98668, Lacombe, Alberta, Canada; 2 Faculty of Science, University of British Columbia 98587 , Vancouver, British Columbia, Canada; 3 Faculty of Science, Red Deer Polytechnic 6827 , Red Deer, Alberta, Canada; University of Strathclyde, Glasgow, United Kingdom

**Keywords:** *Bacillus*, aquaponics, tilapia

## Abstract

We present the 5.7-Mb draft genome sequence of *Bacillus cereus* RAS-11-1 isolated from a tilapia (*Oreochromis niloticus*) aquaponic tank primarily used for educational purposes in a high school in Alberta, Canada. The strain is predicted to carry *trpC* for the biosynthesis of plant growth hormone indole-3-acetic acid (IAA).

## ANNOUNCEMENT

Tilapia aquaponics is a sustainable food production system (1) and provides increased productivity of tilapia (2). Microorganisms in the aquaponics play a significant role in the health of tilapia. Some *Bacillus cereus* strains are foodborne pathogens, but non-pathogenic *B. cereus* can be beneficial (3, 4). A study in China shows that a probiotic *B. cereus* provides health benefits for tilapia by significantly increasing immune enzyme activities in the skin (5). To the best of our knowledge, there has not been any documented study on *B. cereus* in tilapia aquaponics in Canada. Additionally, non-pathogenic *B. cereus* has demonstrated plant growth-promoting properties (PGPs) owing to its ability to produce PGP substances, including indole-3-acetic acid (IAA) (4, 6). Therefore, genomic characterization of our isolated strain is the first step toward understanding the benefits to tilapia aquaponics in Canada.

We isolated *Bacillus cereus* RAS-11-1 from the water of a tilapia tank in an aquaponic system in Lacombe, Alberta, Canada. The aquaponic system primarily serves for educational purposes. A water sample was collected and stored at 4°C until analysis. A 100 µL water sample was plated on Tryptic Soy Agar (TSA; MP Biomedicals, CA) and cultured aerobically at 15°C. A single colony was isolated after 48–72 h incubation. The pure colony was then obtained by re-streaking this single colony on TSA, cultured as above, and then grown in 10 mL Tryptic Soy Broth (TSB; MP Biomedicals, CA) without agitation for DNA extraction.

Cells were pelleted by centrifugation at 5000 × *g* for 10 min at 22°C. Total DNA was extracted from the pellet using Qiagen’s DNeasy Blood & Tissue Kit following the manufacturer’s instructions (Qiagen, Germany) and subjected to whole genome sequencing (WGS) at Génome Québec on the Illumina NovaSeq X Plus sequencing system (150-bp paired-end) using the NEBNext Ultra II DNA Library Prep Kit (New England BioLabs). Quality trimming was performed using fastp (version 0.23.2) (7). The genome was assembled using SPAdes (version 3.15.5) (8). Taxonomic classification was performed by GTDB-Tk (version 2.3.2, classify_wf) (9). Annotation of the genome was carried out using NCBI’s PGAP (version 6.10) (10). Unless otherwise noted, default parameters were used for all software.

A total of 60,342,600 raw reads were generated with genome coverage of 1,533×, and 59,979,992 reads were obtained after filtering. The genome size of RAS-11-1 is 5,700,195 bp, consisting of 53 contigs (N50 = 329.2 kb), 5,606 coding sequences (CDS), 46 tRNAs, and 5 ncRNAs, with an average GC content of 35%. The BLAST-based average nucleotide identity (ANI) (11, 12) is 97.19% identical to that of *B. cereus* ATCC 14579 (assembly version ASM201466v1; GCF_045287585.1/).

The PGAP analysis showed that RAS-11-1 is predicted to carry a 762-bp *trpC* (EC 4.1.1.48; location JBRZRL010000012.1 from nt 22,872 to 23,633) encoding an indole-3-glycerol phosphate synthase involved in tryptophan-dependent IAA synthesis (13, 14).

In conclusion, we have isolated a *B. cereus* strain from a tilapia aquaponic system predicted to carry *trpC* for the biosynthesis of IAA. Future long-read sequencing analyses (15) could further help confirm the genes annotated in RAS-11-1.

## Data Availability

Whole genome shotgun project has been deposited in GenBank under BioProject PRJNA1298282. The WGS FASTQ files of *B. cereus* are available in Sequence Read Archive SRR34822229. The genome assembly was submitted to NCBI under GCA_053413415.1/.
